# Accurate Classification of *NF1* Gene Variants in 84 Italian Patients with Neurofibromatosis Type 1

**DOI:** 10.3390/genes9040216

**Published:** 2018-04-17

**Authors:** Alessandro Stella, Patrizia Lastella, Daria Carmela Loconte, Nenad Bukvic, Dora Varvara, Margherita Patruno, Rosanna Bagnulo, Rosaura Lovaglio, Nicola Bartolomeo, Gabriella Serio, Nicoletta Resta

**Affiliations:** 1 Laboratorio di Genetica Medica, Dipartimento di Scienze Biomediche e Oncologia Umana, Università degli Studi di Bari Aldo Moro, 70124 Bari, Italy; dariacloconte@gmail.com (D.C.L.); nenadbukvic@virgilio.it (N.B.); maritapat@libero.it (M.P.); rosanna.bagnulo@uniba.it (R.B.); lovagliorosaura@gmail.com (R.L.); nicoletta.resta@uniba.it (N.R.); 2 Centro di Malattie Rare, Azienda Ospedaliero-Universitario Policlinico di Bari, 70124 Bari, Italy; patrizia.lastella76@gmail.com; 3 Azienda Ospedaliero-Universitario Policlinico di Bari, 70124 Bari, Italy; dora.varvara@yahoo.it; 4 Sezione di Igiene, Dipartimento di Scienze Biomediche e Oncologia Umana, Università degli Studi di Bari Aldo Moro, 70124 Bari, Italy; nicola.bartolomeo@uniba.it (N.B.); gabriella.serio@uniba.it (G.S.)

**Keywords:** neurofibromatosis type 1, *NF1* gene, variant classification, in silico analysis, splicing

## Abstract

Neurofibromatosis type 1 (NF1) is one of the most common autosomal dominant genetic diseases. It is caused by mutations in the *NF1* gene encoding for the large protein, neurofibromin. Genetic testing of *NF1* is cumbersome because 50% of cases are sporadic, and there are no mutation hot spots. In addition, the most recognizable NF1 clinical features—café-au-lait (CALs) spots and axillary and/or inguinal freckling—appear early in childhood but are rather non-specific. Thus, the identification of causative variants is extremely important for early diagnosis, especially in paediatric patients. Here, we aimed to identify the underlying genetic defects in 72 index patients referred to our centre for NF1. Causative mutations were identified in 58 subjects, with 29 being novel changes. We evaluated missense and non-canonical splicing mutations with both protein and splicing prediction algorithms. The ratio of splicing mutations detected was higher than that reported in recent patients’ series and in the Human Gene Mutation Database (HGMD). After applying in silico predictive tools to 41 previously reported missense variants, we demonstrated that 46.3% of these putatively missense mutations were forecasted to alter splicing instead. Our data suggest that mutations affecting splicing can be frequently underscored if not analysed in depth. We confirm that hamartomas can be useful for diagnosing NF1 in children. Lisch nodules and cutaneous neurofibromas were more frequent in patients with frameshifting mutations. In conclusion, we demonstrated that comprehensive in silico analysis can be a highly specific method for predicting the nature of *NF1* mutations and may help in assuring proper patient care.

## 1. Introduction

Neurofibromatosis type 1 (NF1) is one of the most common genetic disorders, and its estimated prevalence ranges from 1 in 2500 to 1 in 3500 individuals. NF1 is caused by autosomal dominant heterozygous germline mutations in the neurofibromin gene (17q11.2; NM_000267.3). The clinical diagnosis of NF1 is suspected when the following hallmark features are observed in a patient: neurofibromas, cafè-au-lait (CALs) macules, Lisch nodules in the iris, axillary and/or inguinal freckling and subcutaneous or plexiform neurofibromas. In addition to these obvious cutaneous features, optic gliomas, specific bone lesions and learning development problems may also be present. Finally, there is an increased risk for specific malignancies, such as malignant peripheral nerve sheath tumours (MPNSTs) [1,2].

Precise, internationally-recognized guidelines have been set by the NIH (National Institute of Health USA, NIH Consensus Development Conference Statement, 1988) [3]. However, children younger than 12 years of age with no family history of the condition, rarely match the minimum NIH criteria for *NF1*, since the prototypical features may fully develop only after childhood.

The *NF1* gene is a tumour suppressor and encodes for a negative regulator of the Ras guanosine triphosphate (GTP)-ase proteins. The identification of predisposing mutations in the *NF1* gene is complicated by its large size (257 Kb) encompassing 57 constitutive exons and one major alternatively spliced exon. Further challenges to molecular diagnosis are represented by the existence of 15 pseudogenes [4], no mutational hot spots, and a high mutation rate. Therefore, molecular testing of the *NF1* gene is cumbersome, time-consuming and expensive.

Between 2005 and 2015, we performed a mutation search based on a multistep protocol that has been demonstrated to be highly sensitive in detecting *NF1* mutations in patients ascertained through the NIH clinical diagnostic criteria [4,5,6]. This approach included massive complementary DNA (cDNA) sequencing of the *NF1* gene entire coding region encompassed in multiple amplicons as well as multiplex ligand probe amplification (MLPA) to identify whole or partial gene deletions. With the advent of next generation sequencing (NGS), a powerful tool has been added to efficiently analyse the whole genome or multiple predisposing genes in a single run. Since 2016, we have added NGS technology to our diagnostic pipeline. Here, we aimed to evaluate mutation type distributions in our patient cohort and to correlate it with clinical findings.

## 2. Materials and Methods

### 2.1. Patients

NF1 patients were referred to our institute by different clinicians and centres. All subjects signed informed consent prior to the performance of genetic testing and the entire study was approved by the local Ethical Board for Human Experimentation Review (AOU Policlinico di Bari 70124 Bari, Italy). A total of 200 individuals with suspected or clinically diagnosed NF1 were seen in genetic counselling sessions. Two patients were misdiagnosed after NF2 clinical symptoms rather than NF1 symptoms were identified during the pre-test counselling session. Forty-two patients did not meet the NIH minimum criteria for clinical diagnosis and were therefore not screened. The remaining 156 were tested between June 2005 and December 2015 with a combination of cDNA and genomic DNA (gDNA) direct sequencing and MLPA analysis. Starting from January 2016, mutation screening was performed with an NGS-based technique.

### 2.2. DNA and RNA Isolation

DNA was extracted from whole blood using a QIAamp DNA Blood Mini Kit (Qiagen, Venlo, The Netherlands) in accordance with the manufacturer’s instructions. DNA purity and concentration were assessed using NanoDrop 2000 (Thermo Fisher Scientific, Waltham, MA, USA).

Total RNA was isolated from blood collected in a PAXgene Blood RNA Tube IVD (Qiagen) and purified using a PAXgene Blood RNA Kit (Qiagen). RNA samples (1 μg) were reverse transcribed using Maxima reverse transcriptase (Thermo Fisher Scientific, Waltham, MA, USA).

### 2.3. Multiplex Ligand Probe Amplification Analysis

Multiplex Ligand Probe Amplification methodology was used before proceeding with the mutation search to assess whether patients were carrying whole gene or multi-exon deletions. To this end, SALSA kits P081 and P082 (MRC Holland, Amsterdam, The Netherlands) were utilized in accordance with the manufacturer’s instructions. PCR products were separated on an ABI 310 sequencer (Life Technologies, Carlsbad, CA, USA) and data analysed was with Coffalyser software (version 140721.1958, MRC Holland).

### 2.4. Complementary DNA and Genomic DNA Sequencing

*NF1* cDNA was amplified in 22 overlapping fragments according to the conditions described in Valero et al., 2011 [5]. The cDNA alterations were confirmed at the DNA level using genomic primers designed according to several reports [4,7,8,9]. Both cDNA and gDNA PCR products were gel-purified using the GeneClean Kit (Qiagen) and sequenced with an ABI PRISM BigDye terminator sequencing kit v1.1 (Life Technologies) on an ABI 310 Genetic Analyser (Life Technologies). Electrophoretograms were inspected for sequence alterations using the NM_000267.3 transcript as the reference sequence. Relatives were screened only for the identified mutation.

### 2.5. Next Generation Sequencing

Genomic DNA was extracted from peripheral blood cells (PBCs) using the QIAamp Mini Kit (Qiagen, Hilden, Germany) in accordance with the manufacturer’s instructions. DNA was quantified using the Qubit dsDNA HS Assay Kit (Life Technologies) on a Qubit2.0 Fluorometer (Life Technologies, Carlsbad, CA, USA).

The entire coding region of the *NF1* and *SPRED1* genes was sequenced on a personal genome machine (PGM) IonTorrent sequencer. The *NF1* and *SPRED1* custom primers were designed using the AmpliSeq Designer software (Life Technologies, Carlsbad, CA, USA) targeting the complete coding sequence of *NF1* (transcript reference, NM_001042492.2) and *SPRED1* (transcript reference, NM_152594.2) genes. The final custom panel was composed of 116 amplicons divided into 2 primer pools for a total of 20.72 kb of DNA. The estimated panel coverage was 99.52% of the regions of interest (ROI).

Exons left out of the design (i.e., the initial part of exon 5 for the *NF1* gene) were sequenced with the Sanger method. Libraries were constructed from 10 ng of DNA and prepared using the Ion AmpliSeq Library Kit v2.0 (Life Technologies, Carlsbad, CA, USA) in accordance with the manufacturer’s instructions. One of the 16 barcodes of the Ion Xpress Barcode Adapters 1–16 Kit (Life Technologies, Carlsbad, CA, USA) was added to each sample. Amplified libraries were quantified with the Qubit dsDNA HS Assay Kit (Life Technologies, Carlsbad, CA, USA) on a Qubit2.0 Fluorometer (Life Technologies, Carlsbad, CA, USA). Libraries were then pooled in equimolar concentrations and diluted to 100 pM prior to clonal amplification on Ion Sphere Particles using the Ion OneTouch 2 System and Ion PGM Hi-Q View OT2 Kit (Life Technologies, Carlsbad, CA, USA).

Templates were enriched using the Ion OneTouch ES kit (Life Technologies, Carlsbad, CA, USA) and prepared for loading on a 316v2/318v2 chip. Sequencing runs were performed with the Ion PGM Hi-Q View Sequencing Kit (Life Technologies, Carlsbad, CA, USA). Data analysis was performed with Torrent Suite Software v.5.0. (Life Technologies, Carlsbad, CA, USA). Reads were aligned to the hg19 human reference genome from the UCSC (University of California Santa Cruz) Genome Browser and to BED files designed using Ion AmpliSeq Designer. Alignments were verified with Alamut^®^ Visual (version 2.9.0; Interactive Biosoftware, Rouen, France). The mean average read depth, and the percentage of reads on target mapping to the ROI were calculated using the Coverage Analysis plugin (Torrent Suite 5.0.4 software, Life Technologies). For each sample, the ratio of ROI with a minimum coverage of 200× was determined using the amplicon coverage matrix file.

Germline variants in the targeted regions were detected with the variant caller plug-in of the Ion Torrent Suite software (version 5.0.4). The variant caller was run with the default germline and low stringency settings. Variants were filtered with a minimum of 30× coverage, and a sample variant frequency between 30% and 70%. Alamut^®^ Visual was also used for variant interpretation to eliminate strand bias and to reduce false positive calls.

Common single nucleotide variants (minor allele frequency (MAF) > 5%), exonic synonymous variants, and intronic variants were removed from the analysis, while exonic non-synonymous, splice site, and loss-of-function variants were analysed.

### 2.6. Bioinformatic Analyses

All the genetic variants identified were queried by browsing through different databases including the LOVD (Leiden Open Variation Database—http://www.LOVD.nl/NF1), NCBI dbSNP (database of Single Nucleotide Polymorphisms, ClinVar), and Human Gene Mutation Database (HGMD) (http://www.hgmd.org) databases [10]. Reported frequencies of identified variants were verified on the EXAC (http://exac.broadinstitute.org/dbsnp) and 1000 genomes (http://www.internationalgenome.org/data-portal/sample) databases. HGMD and LOVD were also browsed to verify whether a mutation was novel. The putative effects on the NF1 protein were investigated via several prediction algorithms, including SIFT (http://sift.jcvi.org), Polyphen2 (http://genetics.bwh.harvard.edu/pph2/), Mutation Assessor (http:// http://mutationassessor.org/r3/), REVEL (https://sites.google.com/site/revelgenomics/), and CADD (http://cadd.gs.washington.edu).

The impact on mRNA was assessed with the five in silico tools available in the Splicing Module of the variant interpreter software program, Alamut® Visual (Interactive Biosoftware, Rouen, France) with the SpliceSiteFinder (http://www.genet.sickkids.on.ca/~ali/splicesitefinder.html), MaxEntScan (http://genes.mit.edu/burgelab/maxent/Xmaxentscan_scoreseq.html), NNSplice (http://www.fruitfly.org/seq_tools/splice.html) and GeneSplicer (http://www.cbcb.umd.edu/software/GeneSplicer/gene_spl.shtml), and HumanSplicingFinder (http://www.umd.be/HSF/) which combines five different bioinformatics tools. A detailed explanation on the background of scores for each algorithm included in the Alamut Splicing Module can be found at http://www.interactive-biosoftware.com/doc/alamut-visual/2.6/splicing.html.

### 2.7. Statistical Analysis

The differences among age-groups and frameshifting/in-frame groups were evaluated for each parameter under study with Fisher’s exact test. The Chi-Square trend test and Fisher’s exact test were used to compare multiple proportions. When the Fisher’s test was significant, a Tukey’s Multiple Comparison Test was run for post-hoc analysis. The Spearman non-parametric correlation coefficient and Fisher’s exact test were also used to compare the mean splicing scores for each protein predictor (SIFT/Polyphen2/MutationAssessor) with mutation categories. In all comparisons, *p* < 0.05 was considered statistically significant. All analyses were performed using SAS software Version 9.4 for PC.

## 3. Results

### 3.1. Patients’ Screening

**Figure 1:**
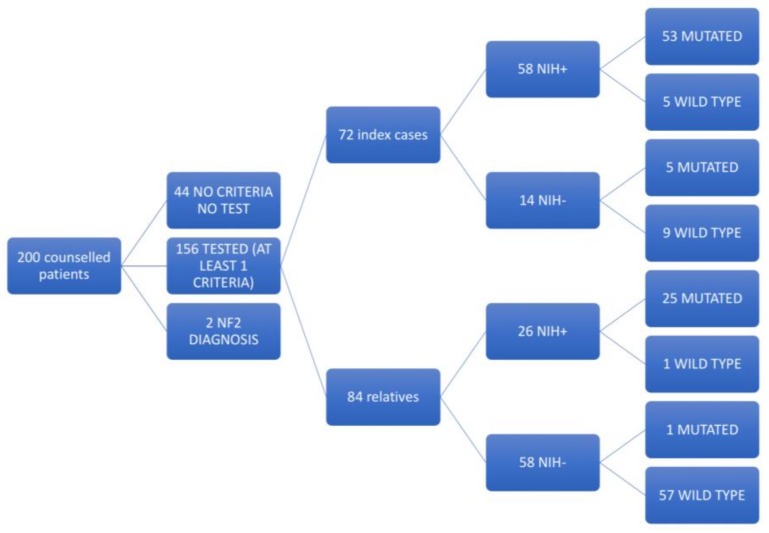
Outline of the patients investigated in this study.

A total of 156 subjects were screened for the detection of causative variants in the *NF1* gene (Figure 1).

Among these subjects, 72 were unrelated index patients, while 84 were investigated after mutation identification since relatives—including 81 first degree relatives—of patients were carrying a pathogenic variant. Twenty-two probands were referred directly to our centre and additional 22 index cases were sent by the local hospital-based Rare Disease Centre. The remaining subjects were referred either by a specialist (eight dermatologists, six neurologists, four paediatricians, two oncologists and one plastic surgeon, endocrinologist and biochemist) or, in five cases, by a family general practitioner. Fourteen of the 72 probands tested did not fulfil the NIH minimum criteria for NF1. Twelve of these 14 NIH-negative probands came from individals under 12 years of age and all had CALs. Thus, it is likely that this subset may include children who had not fully developed NF1 symptoms because of their young age. The remaining two NIH-negative index cases were a 61-year-old man with diffuse neurofibromatosis and a 20-year-old male with twelve CALs but no other NF1 manifestations.

A non-neutral *NF1* variant was identified in five of these NIH-negative probands. In one, the identified variant was subsequently classified as a variant of unknown significance (VUS). In three of the four NIH-negative, mutation-positive index cases, CALs were the only NIH feature present—two of these cases involved individuals under 12 years of age. The remaining NIH-negative mutation-positive patient had no CALs but later disclosed that her brothers had been positively tested for *NF1* mutations six years before. In the genetic counselling session, this subject presented a generic dermatologic referral describing a subcutaneous formation on her thigh. She was also the mother of a ten-year-old boy with one large CAL macule.

Globally, the detection rate in index cases was 80.6%. However, a pathogenic variant was identified in 53 of the 58 NIH-positive index patients, giving an increased detection rate of 91.4%. Twenty-six mutation carriers were younger than 12 years. The clinical features for all tested probands are reported in Table 1.

Genetic testing was extended to 25 siblings, 26 children, 30 parents, and three grandparents to give a total of 84 relatives. Fifty-two first-degree relatives did not fulfil the NIH criteria, and a pathogenic variant was identified only in the son of the 36-year-old woman mentioned previously.

Twenty-six relatives met the minimum clinical criteria for NF1, and only one was negative at *NF1* gene testing. This subject was the 26-year-old sister of a mutation carrier, and she had a single subcutaneous neurofibroma. A family study was possible in 25 cases, resulting in 15 de novo and 10 familial mutations. For the remaining probands, either the first-degree relatives were not available for genetic testing or the results could not conclusively rule out a familial origin of the causative mutation.

Finally, the average age at clinical diagnosis was 10.8 years (range 1–49), while the average age at gene testing was 30.7 years (range 1–71). Thus, we observed an average delay of 19.9 years between the time at diagnosis and molecular analysis.

### 3.2. Molecular Analyses and Classification of Identified Variants

Index cases were analysed via cDNA sequencing and MLPA (*n* = 60) or NGS and MLPA (*n* = 12). All the NIH-positive, cDNA sequencing-negative patients were reanalysed with NGS. In no cases among this subset did we detect a causative variant. We identified a non-neutral variation in the *NF1* gene in 58 patients, and 29 (50%) of these were novel (Table 2). Two mutations were gross deletions encompassing the entire *NF1* gene and exons 12–29, respectively. The remaining 56 were subtle changes distributed among 32 different *NF1* exons. The identified variants were as follows: 20 frameshifting small insertions deletions or indels, 12 nonsense mutations, 14 splicing alterations, seven missense variants, two in-frame deletions, and one in-frame insertion (Table S1).

Only four mutations occurred more than once: c. 2033_2034insC, c.4084C>T (p.1362Arg*), c.4111−2A>T and c.6747_6749delTGT p.(Val2230del). Each was present in two different unrelated probands. Variants causing an unambiguous pathogenic effect on the NF1 protein (frameshifting, nonsense, and nucleotide changes at the invariant positions−2, −1, +1, +2 of intronic sequence) were not investigated further. The remaining missense and splicing variants outside the canonical positions mentioned previously were studied with two different sets of prediction algorithms: SIFT, Polyphen2, MutationAssessor and REVEL were only used for missense mutations. The Alamut Visual^®^ splicing module was used to analyse all variants predicted to alter splicing, and for missense mutations in proximity to the splicing junctions or with conflicting interpretations of the consequences on the encoded protein. Therefore, a total of 11 mutations were investigated with this in silico approach (Table 2).

**Figure 2:**
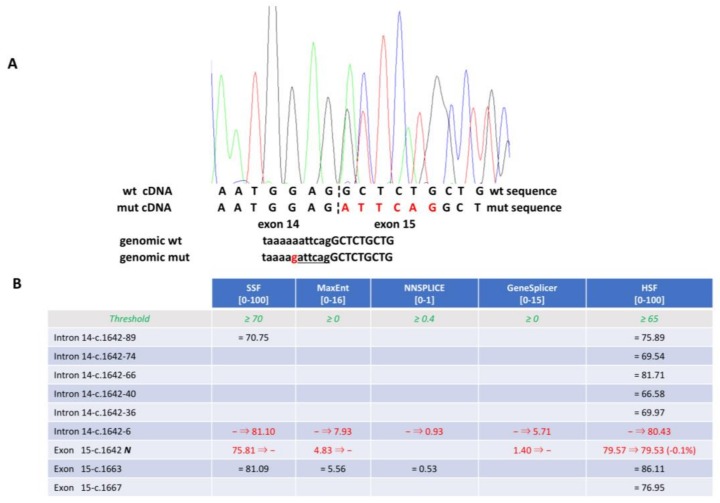
(**A**) Electrophoretogram of the complementary DNA (cDNA) generated from mutation c. 1640-7G>A. Below, the sequences of wild type and mutated cDNA and genomic DNA are presented. The inserted nucleotides are in red in the cDNA sequence. In the genomic sequences, the nucleotide substitution is in red, the nucleotides inserted in the cDNA are underlined. Small letters indicate the intronic sequence. (**B**) Predictions computed from the Alamut Visual splicing module. In the first row, the module’s splicing algorithms are presented; in the second row, the thresholds for the splicing site recognition for each individual algorithm are presented; in the remaining rows, all the putative splicing sites recognized by at least one of the algorithms of the Alamut Visual splicing module are presented. ***N*** indicates the natural splicing site. Green numbers indicate for each algorithm the threshold or range for splicing site recognition; red numbers are used when the computed scores predict splicing alteration; black numbers are used when the splicing site is recognized (above threshold or in the range) but no splicing alteration is forecasted. wt, wild type; mut, mutation.

Three of these 11 variants were intronic substitutions, at positions −7, +3 and +5, respectively. The mutation c.1642–7A>G has never been reported previously, and three of the five Alamut predictors supported an abrogation of the splicing donor site; NNsplice and HSF did not forecast any changes in splicing (Figure 2).

Thus, we decided to perform real time (RT)-PCR with primers encompassing exon 15. The normally-sized PCR product was sequenced to reveal an insertion of six nucleotides that lead to an in-frame insertion of isoleucine and asparagine aminoacids (Figure 2). For mutations c.6756+3A>G and c.5546+5G>A, 4/5 and 5/5 Alamut Visual^®^ splicing predictors forecast a splicing alteration, respectively. In addition, these two variants had already been reported in HGMD as damaging mutations (DM).

The remaining eight variants were putatively missense changes, and six were listed in either the LOVD or the HGMD database. The only novel missense mutation, c.565A>C (p.Lys189Glu), occurred in an 11-year-old patient who had four CALs. This mutation was classified with MutationAssessor with a medium functional impact on the NF1 protein (score 2.095). Analysis of the mutation with SIFT and Polyphen2 classified it as damaging and possibly damaging, respectively (scores 0, and 0.54). To get further information on the causative role for this variant, we used Provean (http://provean.jcvi.org/) as an additional in silico tool. Provean computed a neutral impact on the protein sequence (score −2.23) for this variant. Finally, while CADD predicted a possibly pathogenic role, the REVEL score did not suggest unequivocally a causative role. Thus, we decided to classify this variant as a VUS since no splicing alterations were predicted.

The missense mutations c.2990G>A (p.Arg997Lys), c.4267A>G (p.Lys1423Glu), c.4768C>T (p.Arg1590Trp), and c.6755A>G (p.Lys2252Arg) were at exonic positions −1, −3, −4 and −2 from the splicing donor site, respectively. The mutation c.2990G>A (p.Arg997Lys) was listed in the LOVD database and has been demonstrated to cause a splicing alteration at the RNA level. Indeed, four of the five Alamut predictors suggested a splicing alteration when the criteria used by van Minkelen et al. [11] was applied. The mutation c.4267A>G (p.Lys1423Glu) has been reported 18 times in LOVD and had two of the five predictors that suggest a splicing alteration. Sequencing of the cDNA isolated from the patient carrying this variant showed a normal sequence (data not shown) confirming the results reported from other authors [12]. The c.4768C>T (p.Arg1590Trp) variant was classified as missense in the HGMD database and was not forecasted to disrupt splicing by any of the Alamut predictors; hence, we confirmed its classification as a missense mutation, considering the concordantly damaging scores of both SIFT and Polyphen2. Finally, the c.6755A>G (p.Lys2252Arg) variant—classified as a missense mutation by both LOVD and HGMD mutation databases—was predicted to alter splicing by four of the five Alamut predictors. Polyphen2 predicted this variant to be damaging at the protein level, while both SIFT and MutationAssessor suggested weak/neutral effects for this amino acid change. Unfortunately, the RNA from this patient was not available; nevertheless, we decided to consider this variant a splicing mutation.

### 3.3. Reassessment of Predicted Consequences in Reported Variants

**Figure 3:**
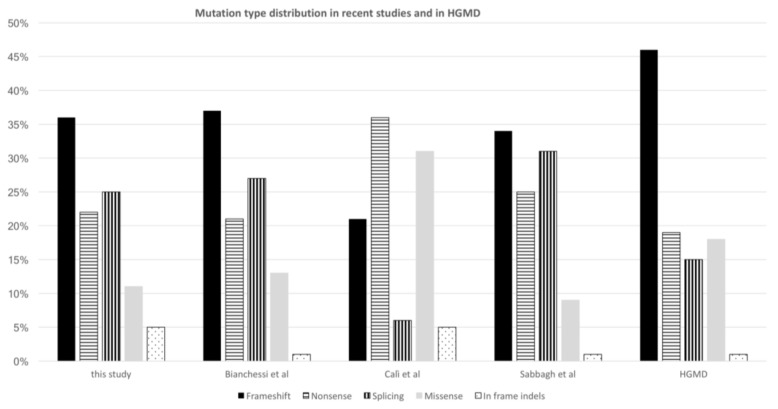
Comparison of mutations type distribution across recent literature and with Human Gene Mutation Database (HGMD). Bianchessi et al. [14], Calì et al. [13], Sabbagh et al. [6].

After classifying all the variants identified in the tested individuals, we compared the mutation type distribution observed in our patients with previously reported cohorts [6,11,13,14]. We found that the ratio of splicing and missense mutations varied widely among these studies and with the one reported in the HGMD (Figure 3).

To investigate whether classification inconsistencies could account for this variability, we decided to analyse all of the HGMD NF1 gene substitutions in exon positions −3, −2, −1 and +1, +2, +3 with respect to the splicing acceptor (SA) and splicing donor (SD) sites using the same in silico analysis detailed previously. We used stricter criteria than Van Minkelen et al. [11], and thus, we considered a variant to be likely altering splicing when (a) at least three of the Alamut^®^ Visual splicing algorithms recognized natural SD or SA sites; and (b) where a ≥10% difference between wild type and mutant sequence scores was observed.

We tested these criteria with nine mutations that were ≤3 nucleotides from the nearest splicing site and were previously demonstrated by Pros et al. [12] to cause splicing alterations in vivo (Table 3). In all cases, regardless of the consequences at the coding level (one nonsense, one neutral, and seven missense), these criteria correctly predicted the splicing outcome. Of the seven mutations with possible missense consequences on the protein, three were predicted to be tolerated with SIFT and were either benign or possibly damaging with Polyphen2 and only one was forecasted to have a high functional impact with MutationAssessor.

Next, we applied the same criteria to 41 *NF1* HGMD variants in exon positions that were ≤3 nucleotides from the adjacent splicing site. Of these 41 variants, 15 were nonsense mutations, 16 were classified as pathogenic missense variants in HGMD (DM), and 10 as possibly pathogenic missense (DM? in HGMD). When analyzed with the Alamut Visual^®^ splicing module, 3/15 of the nonsense mutations, 9/16 of the pathogenic missense variants and 7/10 of the possibly pathogenic missense were predicted to alter splicing according to the previously-mentioned criteria (Table 4).

**Figure 4:**
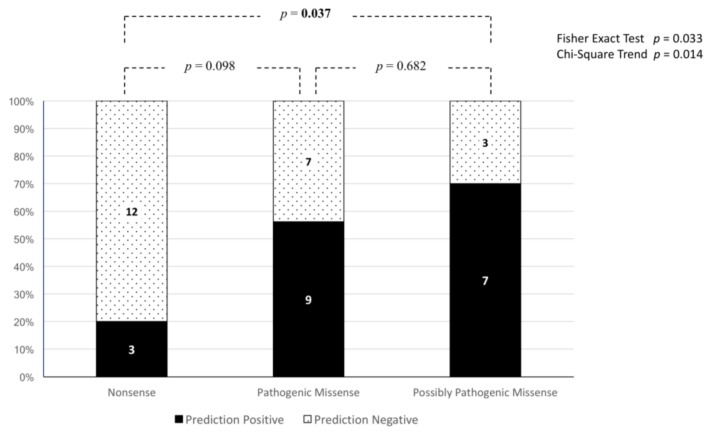
Ratio of HGMD nonsense, pathogenic missense and possibly pathogenic missense variants ≤3 nucleotides from splicing sites predicted to either impact splicing (prediction positive) or have a neutral effect on splicing (prediction negative). In the bars are the absolute numbers for each group. Over the bars, *p*-values for pairwise comparisons, obtained according to Tukey’s method, are represented. In the top-right corner, the global *p*-values for the Fisher’s exact test and for the Chi-Square trend test are presented to compare greater proportions (i.e., more than two).

Therefore, between the variants predicted to putatively disrupt splicing, pathogenic missense and possibly pathogenic missense were significantly more frequent than nonsense mutations (Fisher’s exact test *p* = 0.033). This observation suggests that some variants could have been erroneously considered to be possible pathogenic missense. In fact, we observed a statistically significant trend (Chi-Square *p* = 0.014) for predictions of splicing to be abnormal when less dramatic consequences on the NF1 protein were forecast (Figure 4).

**Figure 5:**
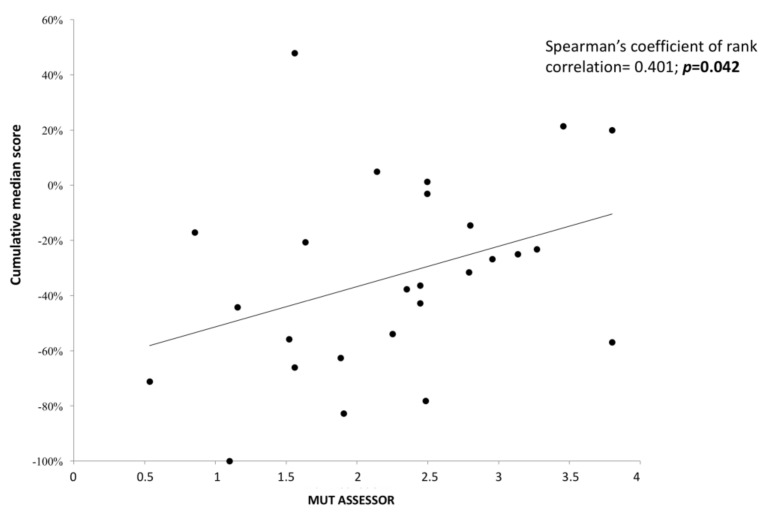
Correlation analysis of the cumulative average score from the five Alamut Visual^®^ splicing predictors with the MutationAssessor scores calculated for 26 HGMD missense mutations ≤3 nucleotides from the nearest splicing site. The data points for this graph are in Table S2.

Also, for the protein predictors used in our study, we found that the MutationAssessor scores for pathogenicity demonstrated a significant correlation with the average scores for defective splicing computed by the Alamut Visual^®^ splicing module (r = 0.401, *p* = 0.042, Figure 5). Since higher values in the Alamut Visual^®^ splicing module are associated with lower/neutral predicted effects on splicing, the correlation was positive.

### 3.4. Genotype–Phenotype Correlations

We correlated the clinical data from the patients with the identified variants subdivided into two categories according to their consequences on the *NF1* coding sequence: (a) **frameshifting,** which included all variants that caused an open reading frameshift (out of frame insertions, deletions, indels, and splicing mutations leading to out of frame exon skipping), and (b) **in-frame**, which comprised missense mutations and all in-frame deletions, insertions, and in-frame exon skippings. A higher frequency of Lisch nodules and neurofibromas was observed in patients carrying frameshifting mutations. Conversely, patients with in frame mutations showed more frequent CALs and freckling. However, these differences did not reach statistical significance (Table 5).

## 4. Discussion

We reported the results of a 10-year study on 200 individuals referred to our unit with a suspected NF1 clinical diagnosis. One hundred fifty-six patients were tested for mutations in the NF1 gene. We obtained an 80.6% detection rate that is comparable to that recently reported by Van Minkelen et al. [11]. When restricted to patients matching the NIH minimal criteria for NF1 diagnosis, the detection rate reached the higher figures reported by many authors [6,13,14]. This finding confirms the importance of expert knowledge on the disease when selecting patients eligible for gene testing.

Since 2016, we have been using targeted next generation sequencing with a panel that also included the *SPRED1* gene. While targeted NGS markedly decreases turnaround time, its amplicon design strategy may miss nucleotide changes localized deeply in the intronic regions. In addition, NGS cannot detect methylation defects that have been demonstrated to inactivate the *NF1* gene in NF1-associated pilocytic astrocytoma [15]. Of the 41 splicing mutations outside canonical splicing positions reported by Evans et al. [16], seven would have been missed by our custom NGS panel because they are not included in the targeted region. However, among our index cases, all patients negative at cDNA-based analysis were also negative with NGS screening.

Of the 72 probands tested, 28 were externally referred. In this group, there were both a higher number of NIH-negative cases (26.7% vs. 7.5%), and a lower mutation detection rate (73.3% vs. 85.7%). Thus, the role of a specialized centre is fundamental to reduce the number of unnecessary gene tests.

We identified five *NF1* pathogenic mutations in NIH criteria-negative index patients. Three of them were <12 years of age, in line with the widely-reported age-dependent onset of various NF1 clinical features. Also, in 12 of the 84 NIH criteria-positive subjects, we failed to identify any pathogenic variant. Seven had only cutaneous manifestations.

CALs were present in all but one of the 26 patients under 12 years of age; eleven had a negative test, showing that CAL spots represent a rather non-specific marker for NF1. This is especially true when patients are externally referred. Indeed, in our experience, descriptions of CALs and less frequently of axillary/inguinal freckling were often incomplete and generic. In many cases, the external referrals reported neither number nor size.

Of the 58 mutations identified, 50% were novel, in agreement with other large NF1 patient cohorts. We observed a slightly higher ratio of splicing mutations compared to that reported in a recent Italian study [14], and to the ratio of splicing variants listed in the HGMD mutation database. These differences encouraged us to investigate whether errors in variants’ classification and/or reporting could be responsible for the observed variations.

In our patients, three of the seven missense mutations identified were most likely predicted to alter splicing. The possibility that more than 30% of missense mutations may disturb splicing has already been proposed by Sabbagh et al. [6]. Also, the frequency of deleterious variants close to splicing sites has been recently reviewed, and substitutions at the first and last two nucleotides in the exons near the splicing acceptor and donor sites, respectively, represented nearly 15% of all variants that negatively influenced splicing [17]. 

Therefore, we used in silico predictions to verify whether the NF1 nucleotide substitutions listed in the HGMD mutation database as missense variants could alter the splicing instead. We focused on 41 missense substitutions localized to positions flanking splicing junctions (i.e., ≤3 nucleotides from natural splicing site). First, we validated this approach using previously reported *NF1* mutations whose association to splicing defects was demonstrated with in vivo RNA studies (Pros et al. 2008, [12]). In this validation set, in silico predictions faithfully recapitulated RNA alterations. Of the 41 HGMD missense substitutions we studied, 19 (46.3%) were predicted to cause splicing deregulation. Conversely, 22 missense substitutions had no predicted consequences on splicing, and 11 were in positions either +3 or −3 from the nearest splicing site. In genetic diseases, these positions are hit by splicing-disrupting changes 20 times less frequently than positions +1/−1 and +2/−2.

If we had used the less stringent criteria adopted by Van Minkelen et al. [11] only 3/28 nucleotide changes positioned ≤2 from nearby splicing sites would have been forecast neutral to the splicing. Two of these changes (c.62T>G and c.62T>C in Table 5) occur at position +2 from the splicing acceptor which is the least conserved position in the splicing junction. The third nucleotide change (c.3707G>A in Table 5, −2 from SD) is a nonsense mutation predicted to slightly increase the splicing site strength.

The first and last two nucleotide positions in the exons are loosely conserved compared with the corresponding intronic positions [18]. Thus, the five algorithms included in the variant analysis software used here were effective in forecasting splicing defects consequent to exonic nucleotide substitutions. Furthermore, variants that putatively affected splicing were most frequent among those with weaker predicted effects at the coding level.

Our approach is coherent with guidelines proposed jointly by the ACMG (American College of Medical Genetics and Genomics) and AMP (Association for Molecular Pathology) that encourage the use of multiple predictors, even when partially relying on the same rationale to get stronger support in classifying variants [19]. Gathering extensive information from different in silico algorithms is thought to lead to more reliable judgment of the variant/disease association. This is increasingly recognized as a valuable tool in the clinic [20,21,22]. These results suggest that all variants near splicing sites that are predicted to alter splicing should be investigated at the RNA level—especially when the forecast consequences on the encoded protein are weak or inconclusive or conflicting.

The variability observed here in the percentage of missense and splicing variants reported in different studies might be partially explained by this misclassification. The advent of massive NGS technology that is essentially DNA-based might have introduced bias in the pathogenicity assessment of missense variants. In contrast, highly sensitive RNA-based approaches have routinely detected a high proportion splicing defect in the *NF1* gene [12,23,24,25].

The elusive search for genotype–phenotype correlations in NF1 patients—even in large patient cohorts—might have been hampered by this mutation classification uncertainty. Of the 41 HGMD variants we investigated with in silico prediction tools, 37 (90%) were in the first half of the NF1 coding sequence compared to 65% of all missense and nonsense substitutions listed in the HGMD database collection. Also, more than two-third of the junction variants studied here were proximal to the splicing donor site, confirming the data of Pros et al. [12].

The correct evaluation of variants’ pathogenicity is important in NF1 patient management. Cognitive and behavioural disorders affect 50–80% of all children with NF1. When these disorders are recognized and treated early, the subjects often have improved academic performance [26,27]. Properly framing the nature of the identified variants may influence NF1 monitoring. Indeed, a recent study found a higher risk for optical glioma and MPNSTs associated with splicing mutations [28].

We could not find any novel genotype–phenotype correlations—the only statistically significant difference was the higher number of adults with neurofibromas. However, the number of neurofibromas has been positively associated with the age of a patient [29]. This was recently confirmed quantitatively [30].

We observed a rather long delay between the time of clinical diagnosis and the age at gene testing. The former was at the beginning of the teenage years, and the latter coincided with the average age at reproduction in the reference population. Although most NF1 symptoms cannot be treated, genetic testing could be helpful in paediatric patients who do not fulfil the NIH criteria for monitoring and intervention should cognitive problems arise.

Only two of our patients had multi-exon deletions—a whole and a partial (exons 12–29) *NF1* gene deletion, respectively. However, none of the two subjects presented the “NF1 microdeletion phenotype” that has been recently described in patients carrying either a large common deletion that removes all the NF1 exons plus additional 14 genes or other types of large deletions including NF1 and a variable number of flanking regions [31,32].

Brain hamartomas were more common than optic gliomas in our subjects. The presence of brain hamartomas has been occasionally reported in NF1 patients [33]. Recently, in a group of Korean NF1 patients, brain hamartomas represented the most common abnormal finding in the brain with a frequency comparable to that observed here [34]. In all our patients with hamartomatous lesions, the NIH criteria were met, and a causative NF1 gene mutation was found in 7/9. All but one were under 12 years of age. Therefore, in our experience, brain hamartomas can be a useful indicator for a NF1 diagnosis. This is particularly true in children where other common NF1 signs might not yet be present or fully evident. The role of paediatric hamartomas in NF1 diagnosis has been recently addressed by various authors as well [35,36,37].

We reported one in-frame deletion, c.6687_6689delTGT (p.Val2230del), present in two independent cases as well as one in-frame insertion caused by intronic substitution, c.1642–7A>G (p.Glu547_Ala548insIleGlu). In contrast to the in-frame deletions reported by Upadhyaya et al. [38] and Calì et al. [13], c.6687_6689delTGT was associated, in our two independent patients, with diffuse neurofibromatosis in an otherwise classical form of NF1. The in-frame insertion was identified in a 2-year-old baby girl with CALs only at referral. However, she developed an optical glioma shortly after gene testing.

Finally, we classified splicing mutations according to their consequences on the reading frame, as previously reported by Sabbagh at al. [6] and Pasmant et al. [39]. Therefore, causative variants were divided in two groups: (a) frameshifting that included nonsense, frameshifting insertion and deletions, and frameshifting exon skipping; and (b) in-frame variants comprising missense, in frame deletions and insertions, and in frame exon skipping. We observed a higher frequency of Lisch nodules in patients carrying frameshifting mutations (22.6% vs. 9.1%), which is consistent with previous observations by Sabbagh et al. [6], and Castle et al. [40]. Similarly, cutaneous neurofibromas were more common in patients with truncating mutations (74.2% vs. 54.5%). However, likely due to the limited size of the population studied, these differences did not reach statistical significance. Accurate classification of previously identified mutations in large datasets will help to evaluate the implication of these findings.

## 5. Conclusions

In conclusion, we here reported 29 novel *NF1* gene mutations—11 detected by NGS. The NGS-based approach was time- and cost-effective compared to the highly-sensitive RNA-based methodology used during our first 8 years of diagnostic testing. However, it may miss variants localized deep in the intronic sequence. The in-silico analysis of HGMD variants performed here suggests that many variants reported as missense are likely to affect splicing instead. This is especially true for variants with inconclusive data on their pathogenicity at the protein level. To this end, the five splicing prediction algorithms included in the variant analysis software could be extremely useful in properly evaluating the causative role of identified variants. An accurate variant classification could lead, in turn, to precise counselling and proper management of NF1 patients and their family relatives.

## Figures and Tables

**Table 1:** Clinical data in mutated probands.

Number of Patients	Total (*n* = 58)	<12 Years (*n* = 26)	>12 Years (*n* = 32)	*p*-Value
**CALs**	**53 (91)**	**25 (96)**	**28 (87)**	**NS**
**Freckling**	**32 (55)**	**15 (58)**	**17 (52)**	**NS**
**Lisch nodules**	**14 (24)**	**5 (21)**	**9 (26)**	**NS**
**Neurofibromas**	**34 (58)**	**11 (42)**	**23 (71)**	**0.03**
**First degree relative**	**8 (13)**	**0 (0)**	**8 (23)**	**0.013**
**Optic glioma**	**3 (5)**	**1 (4)**	**2 (6)**	**NS**
**Bone lesions**	**16 (27)**	**10 (37)**	**6 (19)**	**NS**
**Sphenoid dysplasia**	**3 (5)**	**1 (4)**	**2 (6)**	**NS**
Intellectual disability	7(13)	6 (21)	2 (6)	NS
Amartomas	5 (9)	3 (12)	2 (6)	NS
Deafness incl. neurinomas	5 (9)	0 (0)	5 (16)	NS

In parentheses are the percent of patients with the indicated clinical feature (those in bold are included in the National Institutes of Health (NIH) clinical criteria). *p*-values refer to Fisher’s exact test. NS = statistically not significant.

**Table 2:** In silico analysis of putative missense and splicing mutations.

MUTATION	DIST To NEAREST SS ^a^	SIFT ^b^	POLYPHEN2 ^c^	MUTATION ASSESSOR ^d^	REVEL ^e^	CADD PHRED	SSF ^f^	MES ^f^	NNSPLICE ^f^	GENESPLICER ^f^	HSF ^f^	NOVEL	CLASS
*c.565A>C (p.Lys189Gln)*	*20 (D)*	*D (0.00)*	*PoD (0.54)*	*M (2.095)*	*0.612*	*25*	*No change*	*No change*	*No change*	*No change*	*No change*	*Y*	*MISSENSE*
*c.1642-7A>G*	*7 (A)*	*NE*	*NE*	*NE*	*NE*	*4.1*	*SD abrogation*	*SD abrogation*	*No change*	*SD abrogation*	*No change*	*Y*	*SPLICING*
c.1658A>C (p.His553Pro) *	17 (A)	T (0.06)	PD (0.99)	M (2.395)	0.778	27.4	No change	No change	NA	−14%	No change	CM076335	MISSENSE
c.2990G>A (p.Arg997Lys)	0 (D)	T (0.35)	PoD (0.902)	L (1.535)	0.269	24.7	−13%	−24%	−2%	−79%	−26%	LOVD	SPLICING
*c.3250C>T (p.Pro1084Ser)*	*52 (A)*	*T (0.08)*	*PD (1.00)*	*M (3.235)*	*0.833*	*24.5*	*+3%*	*+13%*	*+11%*	*+137%*	*+1%*	*CM143367*	*MISSENSE*
c.3827G>A (p.Arg1276Gln)	44 (D)	D (0.00)	PD (1.00)	H (3.820)	0.887	34	+1%	SA creation	NA	NA	+1%	CM000802	MISSENSE
c.4267A>G (p.Lys1423Glu)	3 (D)	D (0.00)	PD (1.00)	H (3.80)	0.905	34	−3%	−16%	SD abrogation	No change	−1%	CM920506	SPLICING
c.4768C>T (p.Arg1590Trp)	4 (D)	D (0.01)	PD (1.00)	M (2.72)	0.533	35	No change	No change	+0.2%	NA	No change	CM971051	MISSENSE
c.5546+5G>A	5 (D)	NE	NE	NE	NE	10.3	SD abrogation	SD abrogation	SD abrogation	−14%	−73%	CS076638	SPLICING
c.6755A>G (p.Lys2252Arg)	2 (D)	T (0.75)	PD (0.99)	L (0.84)	0.676	9.4	SD abrogation	−50%	SD abrogation	−6%	−71%	CM143467	SPLICING
c.6756+3A>G	3 (D)	NE	NE	NE	NE	26.5	−6%	−37%	−50%	−11%	−41%	CS031795	SPLICING

* This variant was originally identified from a different laboratory who sent the carrier’s sister DNA to our centre for genetic testing and variant interpretation. In italics are variants identified in patients not fulfilling NIH criteria; ^a^ distance of nucleotides from the nearest splicing site (SS); (D) = donor (A) = acceptor; ^b^ D = damaging, T = tolerated, NE = not evaluated, computed scores are in parentheses; ^c^ PD = probably damaging, PoD = possibly damaging, NE = not evaluated, computed scores are in parentheses; ^d^ L = low functional impact M = medium functional impact, H = high functional impact, computed scores are in parentheses; ^e^ NE = not evaluated; ^f^ SSF = SpliceSiteFinder; MES = MaxEntScan; HSF = HumanSpliceFinder. For all five predictors, SD = splicing donor, SA = splicing acceptor and NA = not assessed. The percentage variation in the strength of the splicing site compared to the normal sequence is reported. LOVD, Leiden Open Variation Database.

**Table 3:** Evaluation of mutations demonstrated to disrupt splicing in vivo by Pros et al. [12], with the five predictors from the Alamut Visual splicing module.

DNA Change	DIST to SS	mRNA Effect	Consequences at Protein Level	SIFT	POLYPHEN	MUT ASSESSOR	SSF	MES	NNPLICE	GENESPLICER	HSF	Predicted to Alter Splicing
**c.58C>T**	−3 SD	r.57_60del4	Gln58*	NE	NE	NE	−5.1%	−23.1%	−28.0%	−35.2%	−2.3%	YES
**c.730G>A**	−1 SD	r.731_888del	Glu244Lys	T (0.45)	B (0.081)	N (−0.60)	−14.0%	−46.6%	SD abrogation	NA	−11.3%	YES
**c.1062G>A**	−1 SD	r.1063_1185del	Lys354Lys	NE	NE	NE	SD abrogation	−79.1%	SD abrogation	NA	−12.5%	YES
**c.1845G>T**	−1 SD	r.1846_2001del	Lys615Asn	T (0.48)	B (0.397)	L (1.845)	−13.4%	−40.1%	−2.6%	SD abrogation	−11.2%	YES
**c.2251G>A**	−1 SD	r.2252_2325del	Gly751Arg	D (0.01)	PD (1.00)	M (2.325)	−13.8%	−49.3%	−29.5%	SD abrogation	−12.0%	YES
**c.2325G>T**	−1 SD	r.2326_2409del	Glu775Asp	T (0.24)	PoD (0.863)	L (1.50)	SD abrogation	−59.3%	SD abrogation	−76.9%	−12.5%	YES
**c.3113G>C**	−1 SD	r.3114_3197del	Arg1038Thr	D (0.02)	PD (0.998)	M (3.27)	−14.5%	−12.0%	−6.4%	SD abrogation	−11.4%	YES
**c.4269G>C**	−1 SD	r.4270_4367del	Lys1423Asn	D (0.00)	PD (1.00)	H (3.80)	SD abrogation	SD abrogation	SD abrogation	NA	−12.8%	YES
**c.5546G>A**	−1 SD	r.5547_5749del	Arg1849Gln	D (0.04)	PD (0.998)	M (3.345)	SD abrogation	−44.1%	SD abrogation	SD abrogation	−12.3%	YES

**Table 4:** In silico analysis of nucleotide substitutions in exon positions −3, −2, −1 and +1, +2, +3.

DNA Change	DIST to SS ^a^	PROTEIN CHANGE	SIFT ^b^	Polyphen2 ^c^	Mutation Assessor ^d^	HGMD CLASS ^e^	SSF ^f^	MES ^f^	NNSPLICE ^f^	GENESPLICER ^f^	HSF ^f^	Predicted to Alter Splicing
c.62T>G	+2 SA	Leu21Arg	D (0.00)	PoD (0.902)	M (2.495)	DM	no change	3.50%	−0.40%	3.90%	−0.60%	NO
c.62T>C	+2 SA	Leu21Pro	D (0.00)	PD (0.99)	M (2.495)	DM	no change	−9.40%	−0.30%	−5%	−0.90%	NO
**c.479G>T**	**−1 SD**	**Arg160Met**	**D (0.01)**	**PD (0.99)**	**M (2.445)**	**DM?**	**−14%**	**SD abrogation**	**−51.90%**	**NA**	**−11.40%**	**YES**
**c.479G>C**	**−1 SD**	**Arg160Thr**	**D (0.04)**	**PD (0.97)**	**M (2.445)**	**DM?**	**−14.80%**	**−94.50%**	**−24.50%**	**NA**	**−11.60%**	**YES**
c.482T>A	+3 SA	Stop	NE	NE	NE	NONSENSE	no change	−2.10%	no change	11.50%	no change	NO
c.482T>G	+3 SA	Stop	NE	NE	NE	NONSENSE	no change	−1.50%	no change	NA	no change	NO
**c.586G>T**	**−1 SD**	**Stop**	**NE**	**NE**	**NE**	**NONSENSE**	**−13.90%**	**−19.10%**	**−1.50%**	**NA**	**−11.50%**	**YES**
c.887A>G	−2 SD	Lys296Arg	T (0.24)	PD (0.97)	L (1.635)	DM?	−9.50%	−12.90%	−0.30%	−75.40%	−5%	NO
**c.1062G>C**	**−1 SD**	**Lys394Asn**	**T (0.07)**	**PD (0.98)**	**L (1.520)**	**DM?**	**SD abrogation**	**−55%**	**−55.10%**	**NA**	**−13%**	**YES**
c.1060A>T	−3 SD	Stop	NE	NE	NE	NONSENSE	−4.70%	−30.00%	−16.40%	NA	−1.20%	NO
c.1639G>T	−3 SD	Stop	NE	NE	NE	NONSENSE	−1.20%	−9.50%	−0.80%	−75.70%	−0.20%	NO
**c.1720A>G**	**−2 SD**	**Ser574Gly**	**T (0.12)**	**PoD (0.954)**	**L (1.560)**	**DM**	**SD abrogation**	**−58.10%**	**SD abrogation**	**NA**	**−6.30%**	**YES**
c.1720A>C	−2 SD	Ser574Arg	T (0.14)	PD (0.99)	M (2.250)	DM	SD abrogation	−9.60%	SD abrogation	NA	−6.20%	NO
**c.1721G>A**	**−1 SD**	**Ser574Asn**	**T (0.12)**	**PD (0.981)**	**L (1.905)**	**DM**	**SD abrogation**	**SD abrogation**	**SD abrogation**	**SD abrogation**	**−13.70%**	**YES**
c.1722C>G	+1 SA	Ser574Arg	T (0.14)	PD (0.994)	L (1.560)	DM	6.70%	27.80%	SA creation	SA creation	4.90%	NO
**c.1846C>G**	**+1 SA**	**Gln616Glu**	**T (1.00)**	**B (0.257)**	**L (1.10)**	**DM**	**NA**	**SA abrogation**	**SA abrogation**	**SA abrogation**	**−11.0%**	**YES**
c.1846C>T	+1 SA	Stop	NE	NE	NE	NONSENSE	−0.60%	−11.00%	−0.10%	SA abrogation	−0.20%	NO
**c.2252G>T**	**+1 SA**	**Gly751Val**	**D (0.03)**	**PD (1.00)**	**M (2.35)**	**DM?**	**−7.50%**	**−41.80%**	**SA abrogation**	**−33.50%**	**−5.40%**	**YES**
c.2407C>T	−3 SD	Stop	NE	NE	NE	NONSENSE	−4.10%	−11%	−0.10%	−79.60%	−2%	NO
**c.2848C>T**	**−3 SD**	**Stop**	**NE**	**NE**	**NE**	**NONSENSE**	**−5%**	**−28.20%**	**−30.20%**	**SD abrogation**	**−2.30%**	**YES**
c.2851G>T	+1 SA	Val951Phe	D (0.01)	PD (0.994)	M (2.800)	DM	−6.90%	−25%	−3.20%	−32.80%	−4.80%	NO
**c.2990G>C**	**−1 SD**	**Arg997Thr**	**T (0.21)**	**PD (0.981)**	**M (2.955)**	**DM?**	**−14.10%**	**−33.50%**	**−0.90%**	**−74.20%**	**−11.30%**	**YES**
**c.2992T>G**	**+2 SA**	**Tyr998Asp**	**T (0.55)**	**PD (0.997)**	**M (3.135)**	**DM?**	**SD abrogation**	**SD abrogation**	**SD abrogation**	**NA**	**SD abrogation**	**YES**
c.3114G>T	+1 SA	Arg1038Ser	D (0.01)	PD (0.981)	M (3.270)	DM	−8.10%	−40.50%	−7.30%	−55%	−5.30%	NO
**c.3197G>A**	**−1 SD**	**Arg1066Lys**	**D (0.00)**	**PoD (0.902)**	**L (1.885)**	**DM**	**−14.80%**	**−85.90%**	**SD abrogation**	**SD abrogation**	**−12.50%**	**YES**
c.3494T>C	−3 SD	Ile1165Thr	D (0.01)	PD (0.96)	M (2.14)	DM?	4.80%	32.70%	22.10%	−37.20%	2.10%	NO
**c.3497G>A**	**+1 SA**	**Gly1166Lys**	**T (0.89)**	**PD (1.00)**	**L (0.855)**	**DM**	**−4.90%**	**−19.50%**	**−21%**	**−36.20%**	**−3.90%**	**YES**
c.3707G>A	−2 SD	Stop	NE	NE	NE	NONSENSE	10.30%	−5.60%	0.20%	17.60%	5.20%	NO
**c.3974G>A**	**−1 SD**	**Arg1325Lys**	**T (0.50)**	**PoD (0.902)**	**N (0.535)**	**DM**	**SD abrogation**	**−72.30%**	**SD abrogation**	**NA**	**−12.30%**	**YES**
**c.3974G>C**	**−1 SD**	**Arg1325Thr**	**D (0.02)**	**PD (0.981)**	**M (2.485)**	**DM**	**SD abrogation**	**SD abrogation**	**SD abrogation**	**NA**	**−12.80%**	**YES**
c.3977T>G	+3 SA	Stop	NE	NE	NE	NONSENSE	no change	1.20%	no change	10.80%	no change	NO
c.4108C>T	−3 SD	Stop	NE	NE	NE	NONSENSE	−4.90%	−19.60%	−2.30%	NA	−2.20%	NO
**c.4269G>T**	**−1 SD**	**Lys1423Asn**	**D (0.00)**	**PD (0.989)**	**H (3.8)**	**DM**	**−15.20%**	**SD abrogation**	**SD abrogation**	**NA**	**−12.70%**	**YES**
c.4267A>C	−3 SD	Lys1423Gln	D (0.00)	PD (0.989)	M (3.455)	DM?	0.40%	3.40%	2%	SD creation	1.10%	NO
c.4267A>G	−3 SD	Lys1423Glu	D (0.00)	PD (0.974)	H (3.8)	DM	−3.30%	−15.80%	SD creation	NA	−0.90%	NO
c.4267A>T	−3 SD	Stop	NE	NE	NE	NONSENSE	−4.60%	−40.30%	SD creation	NA	−1.20%	NO
**c.4367G>C**	**−1 SD**	**Arg1456Thr**	**D (0.00)**	**PD (0.981)**	**L (1.155)**	**DM?**	**−15.20%**	**−49.30%**	**SD abrogation**	**NA**	**−12.60%**	**YES**
c.5203A>T	−3 SD	Stop	NE	NE	NE	NONSENSE	−4.40%	−17%	−5.30%	SD abrogation	−1.20%	NO
**c.6364G>A**	**−1 SD**	**Glu2122Lys**	**D (0.03)**	**PD (0.974)**	**M (2.79)**	**DM**	**−13.70%**	**−22.70%**	**−7.60%**	**SD abrogation**	**−11.30%**	**YES**
c.6640A>T	−2 SD	Stop	NE	NE	NE	NONSENSE	−9.50%	−14.70%	−0.60%	−51.10%	−5.30%	NO
**c.7552G>T**	**−1 SD**	**Stop**	**NE**	**NE**	**NE**	**NONSENSE**	**SD abrogation**	**SD abrogation**	**SD abrogation**	**NA**	**−12.30%**	**YES**

In bold are the NF1 variants predicted to alter splicing. ^a^ distance in nucleotides from the nearest splicing site; ^e^ DM = damaging missense; DM? = possibly damaging missense.

**Table 5:** Genotype–phenotype correlations.

Number of Patients	Frameshifting (*n* = 62)	In-Frame (*n* = 22)	*p*-Value
**CALs**	**55 (88.7)**	**21 (95.4)**	**NS**
**Freckling**	**30 (46.7)**	**14 (63.6)**	**NS**
**Lisch nodules**	**14 (22.6)**	**2 (9.1)**	**NS**
**Neurofibromas**	**46 (74.2)**	**12 (54.5)**	**NS**
**Optic glioma**	**3 (4.2)**	**1 (4.8)**	**NS**
**Bone lesions**	**16 (25.8)**	**6 (28.6)**	**NS**
Intellectual disability	8 (12.9)	2 (9.5)	NS
Hamartomas	6 (9.6)	2 (9.5)	NS
Deafness incl. neurinomas	4 (6.4)	0 (0)	NS

In parentheses are the percentage of patients with the indicated clinical feature (those in bold are included in the NIH clinical criteria). *p*-values were calculated with Fisher’s exact test. NS = statistically not significant.

## Supplementary Materials

The following are available online at http://www.mdpi.com/2073-4425/9/4/216/s1, Table S1: List of non-neutral variants identified in this study, Table S2: Tabular data relative to Figure 5. Supplementary references for in silico tools used in this study.
