# Supplementary material for: Accurate Classification of NF1 Gene Variants in 84 Italian Patients with Neurofibromatosis Type 1
Source: Genes (Basel). 2018 Apr 17;9(4):216. doi: 10.3390/genes9040216 (PMC5924558; doi:10.3390/genes9040216)
Supplement: Supplementary file 1 [file genes-09-00216-s001.zip › genes-280238-SI.docx]

**Supplementary Materials: Accurate Classification of NF1 Gene Variants in 84 Italian Patients with Neurofibromatosis Type 1**

**Alessandro Stella, Patrizia Lastella, Daria Carmela Loconte, Nenad Bukvic, Dora Varvara, Margherita Patruno, Rosanna Bagnulo, Rosaura Lovaglio, Nicola Bartolomeo, Gabriella Serio and Nicoletta Resta**

**Table S1**. List of non-neutral variants identified in this study

| **PATIENT ID** | **EXON^a^** | **DNA CHANGE^a^** | **RNA CHANGE** | **PREDICTED PROTEIN^b^** | **MUTATION TYPE** | **NOVEL^c^** | **VARIANT ID^d^** | **CADD-PHRED SCORES** | **CLASSIFICATION^e^** |
| --- | --- | --- | --- | --- | --- | --- | --- | --- | --- |
| NF1-091 | 2 | c.204+1G>T | r.100_204del | p.(Val34_Met68del) | substitution | N | CS000867 | 25.7 | SPLICING IF |
| NF1-006 | 3 | c.236delT |  | p.(Leu79Tyrfs*6) | deletion | N | CD062204 | 32 | FRAMESHIFT |
| NF1-021 | 3 | c.247_248delC |  | p.(Gln83Serfs*2) | deletion | Y |  | 32 | FRAMESHIFT |
| *NF1-058* | *5* | *c.565A>C* |  | *p.(Lys189Glu)* | *substitution* | *Y* |  | *25* | *MISSENSE* |
| NF1-099 | 8 | c.808C>T |  | p.(Gln270*) | substitution | N | CM154902 | 38 | NONSENSE |
| NF1-030 | 9 | c.1022_1023insTT |  | p.(Ile342Serfs*35) | insertion | N | CI021463 | 32 | FRAMESHIFT |
| NF1-196 | 12 | c.1283_1284insTA |  | p.(Lys428Asnfs*46) | insertion | Y |  | 34 | FRAMESHIFT |
| NF1-144 | 12 | c.1389_1390delinsG |  | p.(Pro464Argfs*9) | indel | Y |  | 34 | FRAMESHIFT |
| *NF1-160* | *15* | *c.1642-7A>G* | *r.1642-6_1642insATTCAG* | *p.(Glu547_Ala548insIleGlu)* | *substitution* | *Y* |  | *4.1* | *IN FRAME INS* |
| NF1-078 | 17 | c.1855A>T |  | p.(Arg619*) | substitution | Y |  | 40 | NONSENSE |
| NF1-044 | 17 | c.1918dupA |  | p.(Thr640Asnfs*9) | insertion | Y |  | 29.1 | FRAMESHIFT |
| NF1-002 | 18 | c.2002-1del GG |  | p.? | deletion | Y |  | 33 | SPLICING FS |
| *NF1-017* | *18* | *c.2033_2034insC* |  | *p.(Ile679Aspfs*21)* | *insertion* | *N* | *CI951961* | *34* | *FRAMESHIFT* |
| NF1-063 | 18 | c.2033_2034insC |  | p.(Ile679Aspfs*21) | insertion | N | CI951961 | 34 | FRAMESHIFT |
| NF1-035 | 18 | c.2156duptT |  | p.(Arg720Profs*6) | insertion | Y |  | 35 | FRAMESHIFT |
| NF1-008 | 21 | c.2481_2482insT |  | p.(Leu828Phefs*3) | insertion | Y |  | 34 | FRAMESHIFT |
| NF1-080 | 21 | c.2629_2630delAT |  | p.(Met877Aspfs*28) | deletion | Y |  | 35 | FRAMESHIFT |
| NF1-053 | 21 | c.2693T>C |  | p.(Leu898Pro) | substitution | N | CM971042 | 26.7 | MISSENSE |
| NF1-163 | 22 | c.2990G>A | r.2851-2990del | p.(Arg997Lys) | substitution | N | LOVD | 24.7 | SPLICING FS |
| NF1-062 | 23 | c.2991-2A>T |  | p.? | substitution | Y |  | 23.9 | SPLICING IF |
| NF1-129 | 25 | c.3228dupA |  | p.(Val1077Serfs*12) | insertion | Y |  | 35 | FRAMESHIFT |
| *NF1-184* | *25* | *c.3250C>T* |  | *p.(Pro1084Ser)* | *substitution* | *N* | *CM143367* | *24.5* | *MISSENSE* |
| NF1-042 | 26 | c.3457_3460delCTCA |  | p.(Leu1153Metfs*4) | deletion | N | CD972351 | 35 | FRAMESHIFT |
| NF1-164 | 28 | c.3721C>T |  | p.(Arg1241*) | substitution | N | CM000729 | 41 | NONSENSE |
| NF1-082 | 28 | c.3827G>A |  | p.(Arg1276Gln) | substitution | N | LOVD | 34 | MISSENSE |
| NF1-103 | 30 | c.3916C>T |  | p.(Arg1326*) | substitution | Y |  | 40 | NONSENSE |
| NF1-049 | 30 | c.4084C>T |  | p.(Arg1362*) | substitution | N | CM971046 | 42 | NONSENSE |
| NF1-137 | 30 | c.4084C>T |  | p.(Arg1362*) | substitution | N | CM971046 | 42 | NONSENSE |
| *NF1-200* | *30* | *c.4110+1G>T* |  | *p.?* | *substitution* | *Y* |  | *26.6* | *SPLICING FS* |
| NF1-089 | 32 | c.4111-2A>T | r.4111-4269del | p.? | substitution | Y |  | 24.7 | SPLICING IF |
| NF1-116 | 32 | c.4111-2A>T | r.4111-4269del | p.? | substitution | Y |  | 24.7 | SPLICING IF |
| NF1-135 | 32 | c.4267A>G | r.4267A>G | p.(Lys1423Glu) | substitution | N | CM920506 | 28.1 | MISSENSE |
| NF1-151 | 33 | c.4338delT |  | p.(Phe1446Leufs*2) | deletion | Y |  | 34 | FRAMESHIFT |
| NF1-114 | 34 | c.4426dupT |  | p.(Ser1476Phefs*5) | insertion | Y |  | 35 | FRAMESHIFT |
| NF1-140 | 34 | c.4441_4442insTTGC |  | p.(Gly1481Valfs*29) | insertion | Y |  | 35 | FRAMESHIFT |
| NF1-143 | 35 | c.4515-1G>A |  | p.? | substitution | N | CS040851 | 26.2 | SPLICING IF |
| NF1-111 | 35 | c.4613G>A |  | p.(Trp1538*) | substitution | N | CM143412 | 42 | NONSENSE |
| NF1-177 | 35 | c.4537C>T |  | p.(Arg1513*) | substitution | N | CM941093 | 40 | NONSENSE |
| NF1-119 | 36 | c.4768C>T |  | p.(Arg1590Trp) | substitution | N | CM971051 | 35 | MISSENSE |
| NF1-156 | 36 | c.4772+1G>T |  | p.? | substitution | Y |  | 26.3 | SPLICING IF |
| NF1-149 | 38 | c.5453delT |  | p.(Ile1818Thrfs*24) | deletion | N | CD982827 | 35 | FRAMESHIFT |
| NF1-198 | 38 | c.5546+5G>A |  | p.? | substitution | N | CS076638 | 10.3 | SPLICING FS |
| NF1-072 | 38 | c.5667dupT |  | p.(Ile1890Tyrfs*2) | insertion | N | LOVD | 35 | FRAMESHIFT |
| NF1-207 | 40 | c.5839delC |  | p.(Arg1947Aspfs*11) | deletion | Y |  | 35 | FRAMESHIFT |
| NF1-121 | 40 | c.5941C>T |  | p.(Gln1981*) | substitution | Y |  | 45 | NONSENSE |
| NF1-131 | 43 | c.6365-1G>T |  | p.? | substitution | Y |  | 24.4 | SPLICING FS |
| NF1-104 | 44 | c.6624G>A |  | p.(Trp2208*) | substitution | N | CM950850 | 44 | NONSENSE |
| NF1-097 | 45 | c.6687_6689delTGT |  | p.(Val2230del) | deletion | Y |  | 35 | IN FRAME DEL |
| NF1-124 | 45 | c.6687_6689delTGT |  | p.(Val2230del) | deletion | Y |  | 42 | IN FRAME DEL |
| *NF1-108* | *45* | *c.6709C>T* |  | *p.(Arg2237*)* | *substitution* | *N* | *CM000815* | *23.2* | *NONSENSE* |
| NF1-197 | 45 | c.6755A>G |  | p.(Lys2252Arg) | substitution | N | CM143467 | 9.4 | MISSENSE |
| NF1-088 | 45 | c.6756+3A>G |  | p.? | substitution | N | CS031795 | 26.5 | SPLICING FS |
| NF1-171 | 47 | c.6999+1G>T |  | p.? | substitution | N | CS1512941 | 23.5 | SPLICING IF |
| NF1-077 | 49 | c.7127-1G>C |  | p.? | substitution | Y |  | 42 | SPLICING IF |
| NF1-076 | 54 | c.7846C>T |  | p.(Arg2616*) | substitution | N | CM950853 | 36 | NONSENSE |
| NF1-162 | 55 | c.7981delC |  | p.(His2661Metfs*57) | deletion | Y |  | 42 | FRAMESHIFT |
| NF1-070 | 1-58 | Deletions of exons 1-58 |  | p.? | gross del | N |  |  | GROSS REARR |
| NF1-133 | 12-29 | Deletions of exons 12-29 |  | p.? | gross del | Y |  |  | GROSS REARR |

In italics, the variants identified in the patients not fulfilling the NIH clinical criteria. For all the variants listed there was no 1000genome frequency available while in the ExAC database the variants with frequency were the following (frequency in parenthesis): c.204+1G>T (4.065e-6), c.3827G>A (3.611e-6), c.4084C>T (4.063e-6), c.4537C>T (8.126e-6), c.4768C>T (4.067e-6).

a. reference sequence NM_000267.3,

b. reference sequence NP_000258.1

c. In this column is indicated whether the identified variant is novel (Y=yes) or has been previously reported (N=no)

d. Variant ID as reported in HGMD

e. FRAMESHIFT=all out of frame insertions, deletions or indels; SPLICING IF=mutation predicted to lead to in frame exon skipping; SPLICING FS=mutation predicted to lead to out of frame exon skipping

**Table S2**. Tabular data relative to figure 5

| **MUTATION** | **PROTEIN** | **MUTASSESSOR SCORE** | **ALAMUT VISUAL CUMULATIVE AVERAGE SCORE** |
| --- | --- | --- | --- |
| c.62T>C | Leu21Pro | 2,495 | -3% |
| c.62T>G | Leu21Arg | 2,495 | 1% |
| c.479G>T | Arg160Met | 2,445 | -43% |
| c.479G>C | Arg160Thr | 2,445 | -36% |
| c.887A>G | Lys296Arg | 1,635 | -21% |
| c.1062G>C | Lys394Asn | 1,52 | -56% |
| c.1720A>C | Ser574Arg | 2,25 | -54% |
| c.1720A>G | Ser574Gly | 1,56 | -66% |
| c.1720A>C | Ser574Arg | 1,56 | 48% |
| c.1721G>A | Ser574Asn | 1,905 | -83% |
| c.1846C>G | Gln616Glu | 1,1 | -100% |
| c.2252G>T | Gly751Val | 2,35 | -38% |
| c.2851G>T | Val951Phe | 2,8 | -15% |
| c.2990G>C | Arg997Thr | 2,955 | -27% |
| c.2992T>G | Tyr998Asp | 3,135 | -25% |
| c.3114G>T | Arg1038Ser | 3,27 | -23% |
| c.3197G>A | Arg1066Lys | 1,885 | -63% |
| c.3494T>C | Ile1165Thr | 2,14 | 5% |
| c.3497G>A | Gly1166Lys | 0,855 | -17% |
| c.3974G>A | Arg1325Lys | 0,535 | -71% |
| c.3974G>C | Arg1325Thr | 2,485 | -78% |
| c.4269G>T | Lys1423Asn | 3,8 | -57% |
| c.4267A>C | Lys1423Gln | 3,455 | 21% |
| c.4267A>G | Lys1423Glu | 3,8 | 20% |
| c.4367G>C | Arg1456Thr | 1,155 | -44% |
| c.6364G>A | Gly751Val | 2,35 | -38% |

The values in this table are those present in table 5 for MutAssessor. The cumulative Alamut Visual scores are the average between the scores returned by the five independent algorithms included in Alamut Splicing Module. When algorithms predicted a splicing site abrogation or a splicing site creation these were considered equal to -100% and 100%, respectively.

**SUPPLEMENTARY REFERENCES FOR ALGORHITMS INTERPRETATION**

1. Kircher M, Witten DM, Jain P, O'Roak BJ, Cooper GM, Shendure J. A general framework for estimating the relative pathogenicity of human genetic variants*.* *Nat Genet*. **2014,** 46, 310-315
2. Ioannidis NM, Rothstein JH, Pejaver V, Middha S, McDonnell SK, Baheti S, Musolf A, Li Q, Holzinger E, Karyadi D, Cannon-Albright LA, et al*.* [**REVEL**: An Ensemble Method for Predicting the Pathogenicity of Rare Missense Variants.](https://www.ncbi.nlm.nih.gov/pubmed/27666373) *Am J Hum Genet*. **2016**, 99:877-885
3. Vaser R, Adusumalli S, Ngak Leng S, Sikic M, & Ng PC. SIFT missense predictions for genomes. *Nat Protocols* **2016**, 11,1-9
4. Adzhubei I, Jordan DM, Sunyaev SR. Predicting functional effect of human missense mutations using PolyPhen-2. *Curr Protoc Hum Genet*. 2013 Jan; Chapter 7, Unit7.20
5. Cartegni L, Wang J, Zhu Z, Zhang MQ, Krainer AR. ESEfinder: A web resource to identify exonic splicing enhancers. *Nucleic Acids Res* **2003**, 31, 3568-71
6. Desmet FO, Hamroun D, Lalande M, Collod-Béroud G, Claustres M, Béroud C. Human Splicing Finder: an online bioinformatics tool to predict splicing signals. *Nucleic Acid Research* **2009,** 37, e67.
7. Fairbrother WG, Yeh RF, Sharp PA, Burge CB. Predictive identification of exonic splicing enhancers in human genes. *Science* **2002**, 297, 1007-13
8. Hellen Splice Site Tools: A Comparative Analysis Report. NGRL Manchester Report 2009.
9. Houdayer C, Caux-Moncoutier V, Krieger S, Barrois M, Bonnet F, Bourdon V, Bronner M, Buisson M, Coulet F, Gaildrat P, et al. Guidelines for splicing analysis in molecular diagnosis derived from a set of 327 combined in silico/in vitro studies on BRCA1 and BRCA2 variants. *Hum Mutat*. **2012**, 33, 1228-38.
10. Pertea M, Lin X, Salzberg SL. GeneSplicer: a new computational method for splice site prediction. *Nucleic Acids Res* **2001**, 29, 1185-90
11. Raponi, M., Kralovicova, J., Copson, E., et al. Prediction of single-nucleotide substitutions that result in exon skipping: identification of a splicing silencer in BRCA1 exon 6. *Hum Mutat*. **2011**, 32, 436-444.
12. Reese MG, Eeckman FH, Kulp D, Haussler D. Improved Splice Site Detection in Genie. J Comp Biol **1997**, Fall,4, 311-23
13. Yeo G, Burge CB. Maximum entropy modeling of short sequence motifs with applications to RNA splicing signals. *J Comput Biol* **2004**, 11, 377-94
14. Zhang MQ. Statistical features of human exons and their flanking regions. *Hum Mol Genet* **1998**, 7, 919-32
